# Establishment and epidemiological investigation of a dual fluorescent qPCR assay for *Pasteurella multocida* and *Salmonella* in yaks in the Tibetan Autonomous Prefecture of Garzê, China

**DOI:** 10.3389/fcimb.2025.1599817

**Published:** 2025-06-04

**Authors:** Yao Pan, Qingting Yu, Qi Wang, Qiang Li, Wei Tian, Lingxiang Xin, Xing Hu, Haiyue Xiao, Yuanjie Liu, Luo Rong Deng Zhu, Lan Lan, Liangquan Zhu, Jianping Wu

**Affiliations:** ^1^ Animal Husbandry Science Institute of Ganzi Tibetan Autonomous Prefecture, Kangding, China; ^2^ China Institute of Veterinary Drug Control, Beijing, China

**Keywords:** yak, *Pasteurella multocida*, *Salmonella*, duplex real-time fluorescence PCR, detection

## Abstract

**Introduction:**

Yaks serve as a vital economic and ecological resource in high-altitude regions, but it faces significant health challenges from various pathogens. Among these, *Pasteurella multocida* and *Salmonella* are critical pathogens that contribute to severe diseases.

**Methods:**

A duplex real-time fluorescence quantitative PCR assay was developed to simultaneously detect *Pasteurella multocida* and *Salmonella*. The species-specific genes *kmt1* and *invA* were selected as target regions for primer and probe design. Following rigorous optimization, a duplex assay was established. Recombinant plasmids were constructed to serve as standards for generating standard curves. The detection thresholds were determined using SPSS statistical analysis and receiver operating characteristic curve methods. Furthermore, the assay’s sensitivity, specificity, stability, and clinical applicability were evaluated.

**Results:**

The established assay demonstrated high sensitivity, with detection limits of 100 and 10 copies for pMD-kmt1 and pMD-invA, respectively. No cross-reactivity was observed with six pathogens, including *Mycoplasma bovis*, infectious bovine rhinotracheitis virus and others. The standard curves showed strong linearity, with coefficients of determination of 0.995 and 0.998, and amplification efficiencies of 103.37% and 103.47% for pMD-kmt1 and pMD-invA, respectively. No interference was observed between high- and low-concentration templates during simultaneous detection. The intra- and inter-assay coefficients of variation ranged from 0.23% to 1.51%. Detection thresholds were determined to be cycle threshold values of 41.5 for *P. multocida* and 40.0 for *Salmonella*. Clinical evaluation was performed on 226 samples collected from yaks in seven counties of Ganzi Prefecture, Sichuan Province, China. The single infection rates of *P. multocida* and *Salmonella* were 20.35% (46/226) and 38.50% (87/226), respectively, while the co-infection rate was 6.19% (14/226).

**Discussion:**

This study successfully established a duplex real-time fluorescence PCR assay that enables the simultaneous detection of *P. multocida* and *Salmonella* with high sensitivity, specificity, and efficiency. The assay offers a reliable and rapid diagnostic tool that is particularly suited for clinical and epidemiological investigations in yak populations.

## 1 Introduction

The yak (*Bos grunniens*), a vital livestock species in high-altitude regions, is widely distributed across the Qinghai-Tibet Plateau and surrounding areas, owing to its exceptional adaptability to cold and hypoxic environments (Tiwari et al., 2024). Yaks serve as a pillar of the local livestock economy and ecosystem, providing essential resources such as meat, milk, and hides to herders (Ai et al., 2024). However, the increasing stocking density of yak populations in recent years has markedly elevated the risk of disease outbreaks and transmission, including bovine pasteurellosis and bovine paratyphoid (Mo et al., 2024; Ran et al., 2024).

*Pasteurella multocida* (*P. multocida*), a Gram-negative bacterium, is classified into five capsular serogroups (A, B, D, E, and F) and is a major causative agent of bovine respiratory diseases (serogroup A) and hemorrhagic septicemia (serogroups B and E) (Wang H. et al., 2023). *Salmonella*, another Gram-negative bacterium with over 2,600 serotypes, is an important pathogen responsible for septicemia and gastroenteritis (Craig et al., 2024; Ran et al., 2024). Commonly present in the intestinal tracts of animals and in environmental reservoirs such as water and soil, *Salmonella* spreads primarily through contaminated feed, water, or direct contact (Ayuti et al., 2024). *P. multocida* and *Salmonella* are opportunistic pathogens, and healthy animals can act as asymptomatic carriers under specific conditions (Mohler et al., 2009; Shehta et al., 2022; Wang H. et al., 2023). In the harsh plateau environment, characterized by hypoxia, extreme cold, and limited forage availability, yaks experience significant physiological stress, which predisposes them to infections by these pathogens (Piorunek et al., 2023a; Wang H. et al., 2023; Ran et al., 2024). Furthermore, *P. multocida* and *Salmonella* have zoonotic potential, posing threats to human health (Piorunek et al., 2023a; Piorunek et al., 2023b; Grote et al., 2024).

Current etiology diagnostic methods for *P. multocida* and *Salmonella* include bacterial isolation and identification and polymerase chain reaction (PCR) (Islam et al., 2023; Mahboob et al., 2023; Bahr and Abdel, 2024; Shaukat et al., 2024). While bacterial isolation is considered the “gold standard,” it is labor-intensive, time-consuming, and requires specialized laboratory infrastructure (Islam et al., 2023). Traditional PCR is limited by low sensitivity and specificity which reduces its efficiency and accuracy (Bahr and Abdel, 2024).

In contrast, duplex real-time fluorescence quantitative PCR is a highly efficient diagnostic approach that combines speed, sensitivity, specificity, and quantification capabilities (Zheng et al., 2020; Lv et al., 2022). This method enables the simultaneous detection of multiple pathogens in a single assay (Zheng et al., 2020; Lv et al., 2022). Its simplicity and reliability make it an ideal tool for pathogen detection and disease control. Developing a duplex real-time fluorescence quantitative PCR assay for the simultaneous detection of *P. multocida* and *Salmonella* would significantly enhance diagnostic sensitivity and specificity, facilitate the rapid identification of infected animals, and provide robust technical support for the effective prevention and control of these diseases in yak populations.

## 2 Materials and methods

### 2.1 Nucleic acid of bacteria and viruses

The nucleic acids of *Pasteurella multocida* (Serotype A), *Pasteurella multocida* (Serotype B), *Pasteurella multocida* (Serotype E), *Salmonella Dublin, Salmonella typhimurium, Mycoplasma bovis, Escherichia Coli* O157, *Staphylococcus aureus*, *Pseudomonas aeruginosa*, Infectious Bovine Rhinotracheitis Virus (IBRV), Clostridium perfringens were from the China institute of Veterinary Drug Control.

### 2.2 Primer and probe design

**Figure 1:**
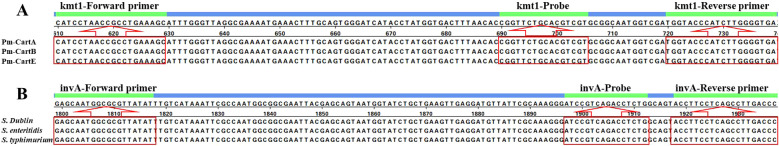
Sequence alignment of target genes of *P. multocida* **(A)** and *Salmonella* **(B)**.

The *kmt1* gene of *P. multocida* (GenBank ID: CP033599.1) and the *invA* gene of *Salmonella* (GenBank ID: CP060494.1) were selected as target sequences. Gene sequences were aligned using BLAST, and conserved regions were identified using MegAlign software (**Figure 1**). Primers and probes were designed with Primer Express 3.0.1 software and synthesized by Tsingke Biotechnology Co., Ltd. Select specific and conserved primers and probes through experiments (**Table 1**). The 5’ ends of the probes for *P. multocida* and *Salmonella* were labeled with FAM and Cy5, respectively, while an MGB quencher was added to the 3’ end. The working concentrations of primers and probes are both 10 μ M.

**Table 1:** Fluorescence quantitative PCR primers and probes.

Pathogens	Gene	Sequence (5′-3′)	Product size (bp)
*P. multocida*	*kmt1*	F: CATCCTAACCGCCTGAAAGC R: TCACCCCAAGATGGGTACCA Probe: FAM-CGGTTCTGCACGTCGT-MGB	130
*Salmonella*	*invA*	F: GGAGCAATGGCGCGTTATAT R: GGGTCAAGGCTGAGGAAGGT Probe: VIC-ATCCGTCAGACCTCTG-MGB	140

### 2.3 Reaction system optimization

The primer and probe concentrations (ranging from 0.1 μM to 0.5 μM), annealing temperatures (ranging from 56°C to 62°C) and number of cycles (40×, 45×, 50×) for a single-fluorescence quantitative PCR assay were optimized using both the matrix and single-variable methods. The optimal reaction conditions were selected based on criteria of low Ct values and high amplification efficiency. Subsequently, the reaction system and program for the duplex fluorescence quantitative PCR assay were further optimized using the parameters established for the single-fluorescence PCR assay.

### 2.4 Standard curve and sensitivity testing

The target sequences of *P. multocida* and *Salmonella* were synthesized to construct recombinant plasmid standards, pMD-Pm and pMD-SE, respectively. The concentrations of the recombinant plasmid standards were measured and converted to copy numbers using a standard formula. Both plasmid standards were adjusted to a final concentration of 1 × 10^10^ copies/μl and then mixed in equal volumes to obtain a combined concentration of 0.5 × 10^10^ copies/μl. A 10-fold serial dilution was performed from 0.5 × 10^10^ copies/μl to 0.5 × 10^10^ copies/μl. The optimized duplex fluorescence quantitative PCR method was applied to detect plasmids at various concentrations, generating a standard curve and determining the limit of detection.

### 2.5 Specificity testing

DNA from *P. multocida* (serotype A, B, and E), *Salmonella Dublin*, and *Salmonella typhimurium* was used as positive controls, while sterile water served as the negative control. The optimized dual-fluorescence quantitative PCR method was employed to detect the nucleic acids of *Mycoplasma bovis*, *Escherichia coli* O157, *Staphylococcus aureus*, *Pseudomonas aeruginosa*, IBRV, and *Clostridium perfringens*, in order to assess the specificity of the method.

### 2.6 Anti-interference and stability testing

After cross-mixing the plasmid standards at 10^8^ and 10^3^ copies/µL, the mixture was used as the template for detection using the optimized dual-fluorescence quantitative PCR method. To evaluate whether high-concentration plasmids could interfere with the amplification of low-concentration plasmids. The reaction components, including enzymes, buffers, primers, and probes, were assembled under optimal conditions. Three gradient-positive control plasmid standards (10^7^, 10^5^, and 10³ copies/µL) were stored at -20°C. To assess the method’s reproducibility and stability, these controls were tested every two weeks.

### 2.7 Critical value analysis

This method was applied to test 50 negative samples and 50 positive samples. These 100 samples were tested using the standards issued by Sichuan Province, China “Diagnostic Technical Specification for Yak Pasteurellosis Disease” (Standard number: DB51/T 2298-2016) and “Technical Specification for Isolation and Identification of Salmonella from Yak Sources” (Standard number: DB51/T 1836-2014) recommend methods for testing to determine the background of the sample. Statistical analysis was performed using SPSS, and a Receiver Operating Characteristic (ROC) curve was generated to determine the sensitivity (Se), specificity (Sp), and area under the curve (AUC) of the detection method. The Youden index (Se+Sp−1) was calculated, with the detection threshold corresponding to the value yielding the highest Youden index (Barraclough, 2012; Krupinski, 2021; Pantoja-Galicia et al., 2021).

### 2.8 Clinical sample testing

A total of 226 clinical samples (nasal swabs, rectal swabs, feces, and serum) of yaks were collected from various regions of Garze Tibetan Autonomous Prefecture, Sichuan Province from July 2023 to December 2024, including 22 samples from Jiulong County, 15 samples from Garze County, 59 samples from Daofu County, 31 samples from Liuhe County, 28 samples from Litang County, 37 samples from Yajian County, and 34 samples from Seda County. These samples were collected from healthy yaks, yaks with respiratory symptoms, and calves with diarrhea symptoms. Nucleic acids were extracted following the manufacturer’s instructions for commercial kits (TIANGEN, Beijing). The nucleic acids of these samples were then simultaneously detected using the dual-fluorescence quantitative PCR method established in this study and a previously reported single-fluorescence quantitative PCR method (Demirci et al., 2019; Otten et al., 2024). The concordance between the two methods was compared.

## 3 Results

### 3.1 Optimization of dual fluorescence quantitative PCR conditions

**Figure 2:**
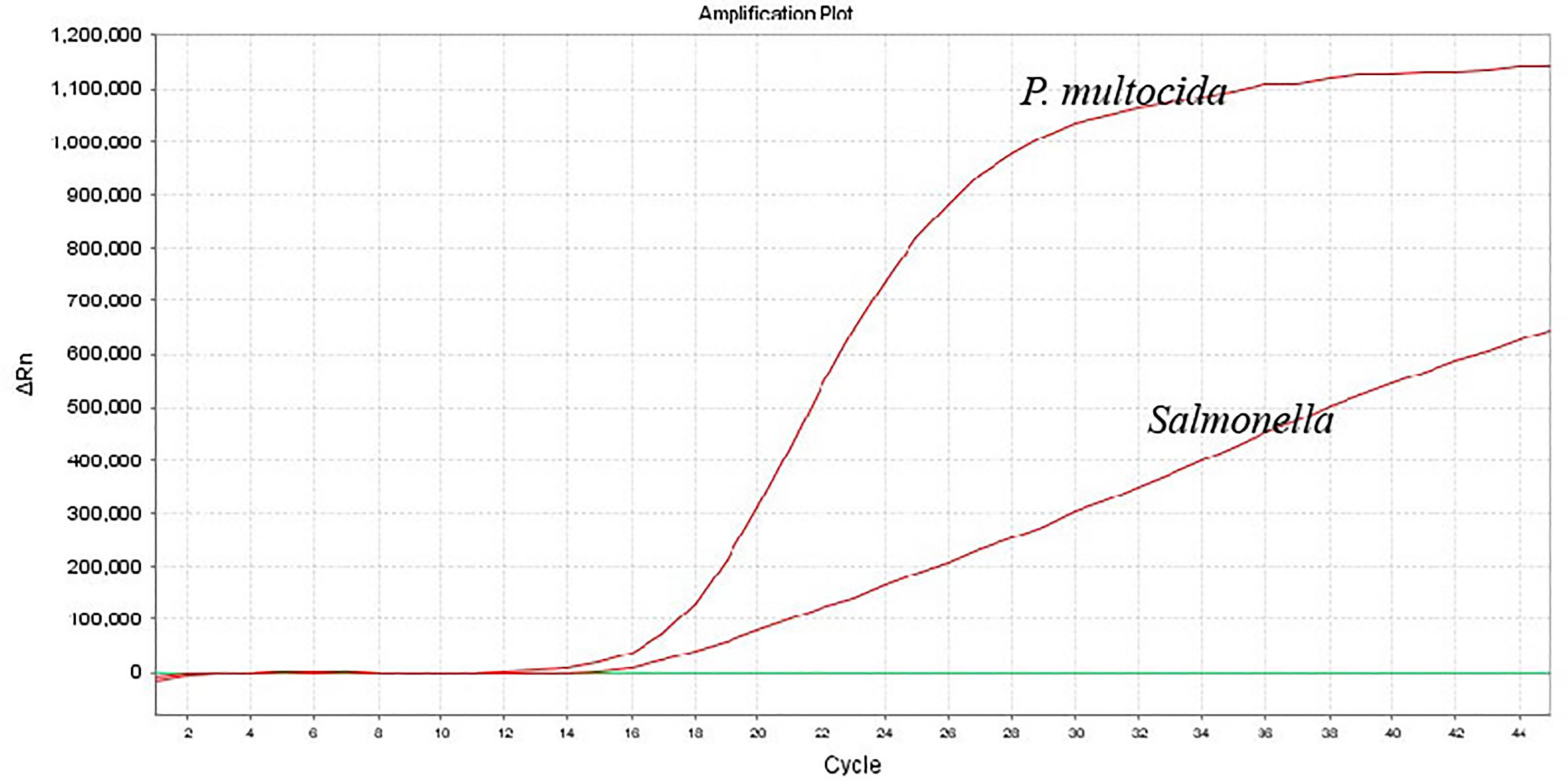
Amplification results of the dual-fluorescence quantitative PCR method.

To enhance the amplification efficiency of the dual-fluorescence quantitative PCR, the annealing temperature, number of cycles, as well as the primer and probe concentrations, were optimized using the single-variable control method. The dual fluorescence quantitative PCR assay utilized 25 μL reaction mixture, consisting of 12.5 μL of 2 × Probe qPCR Mix (Takara, Dalian), 0.5 μM of kmt1-forward primer and kmt1-reverse primer, 0.2 μM of kmt1-probe, 0.45 μM of invA-forward primer and invA-reverse primer, 0.3 μM of invA-probe, 2.0 μL of template, add ddH_2_O to a final volume of 25 µL. When the number of cycles is 45, the amplification efficiency of this method is high and there are no non-specific reactions. The amplification parameters were 95°C for 30 s, and then 45 cycles of 95°C for 5 s and 60°C (annealing temperature and extension temperature) for 34 s. It can specifically amplify *P. multocida* and *Salmonella* (**Figure 2**).

### 3.2 Standard curves and sensitivity

**Figure 3:**
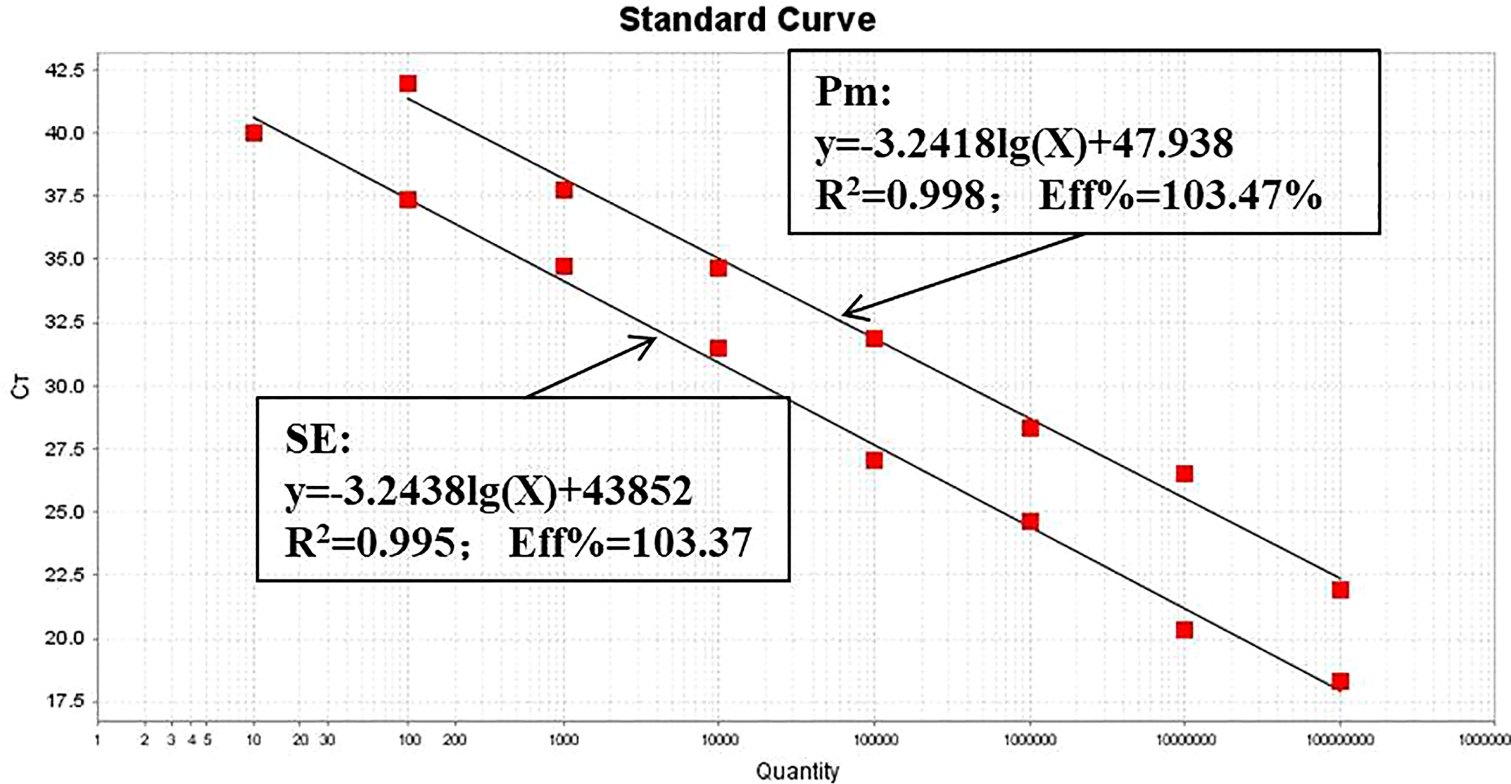
Standard curves of recombinant plasmid standards of *P. multocida* and *Salmonella*.

**Figure 4:**
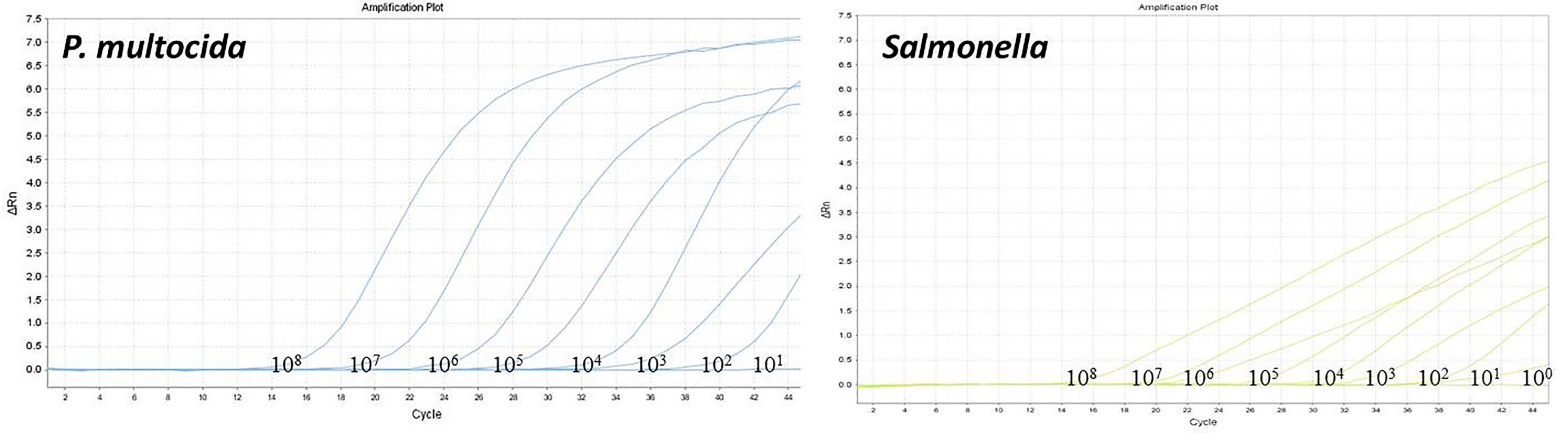
Results of sensitivity of the dual-fluorescence quantitative PCR method.

The optimized dual-fluorescence quantitative PCR method was used to amplify standard plasmids at different gradient concentrations. The standard curve of *P. multocida* was established using 10^8^ to 10^2^ copies, the standard curve of *Salmonella* was constructed using 10^8^ to 10^1^ copies. Both standard curves demonstrated strong linear relationships, with R² values of 0.995 and 0.996, respectively, both exceeding 0.990. The amplification efficiencies (Eff) were high, at 103.47% and 103.37%, respectively (**Figure 3**). The limit of detection of the dual-fluorescence quantitative PCR method for the recombinant plasmid standards of *P. multocida* and *Salmonella* were 100 copies and 10 copies, respectively (**Figure 4**).

### 3.3 Results of specificity

**Figure 5:**
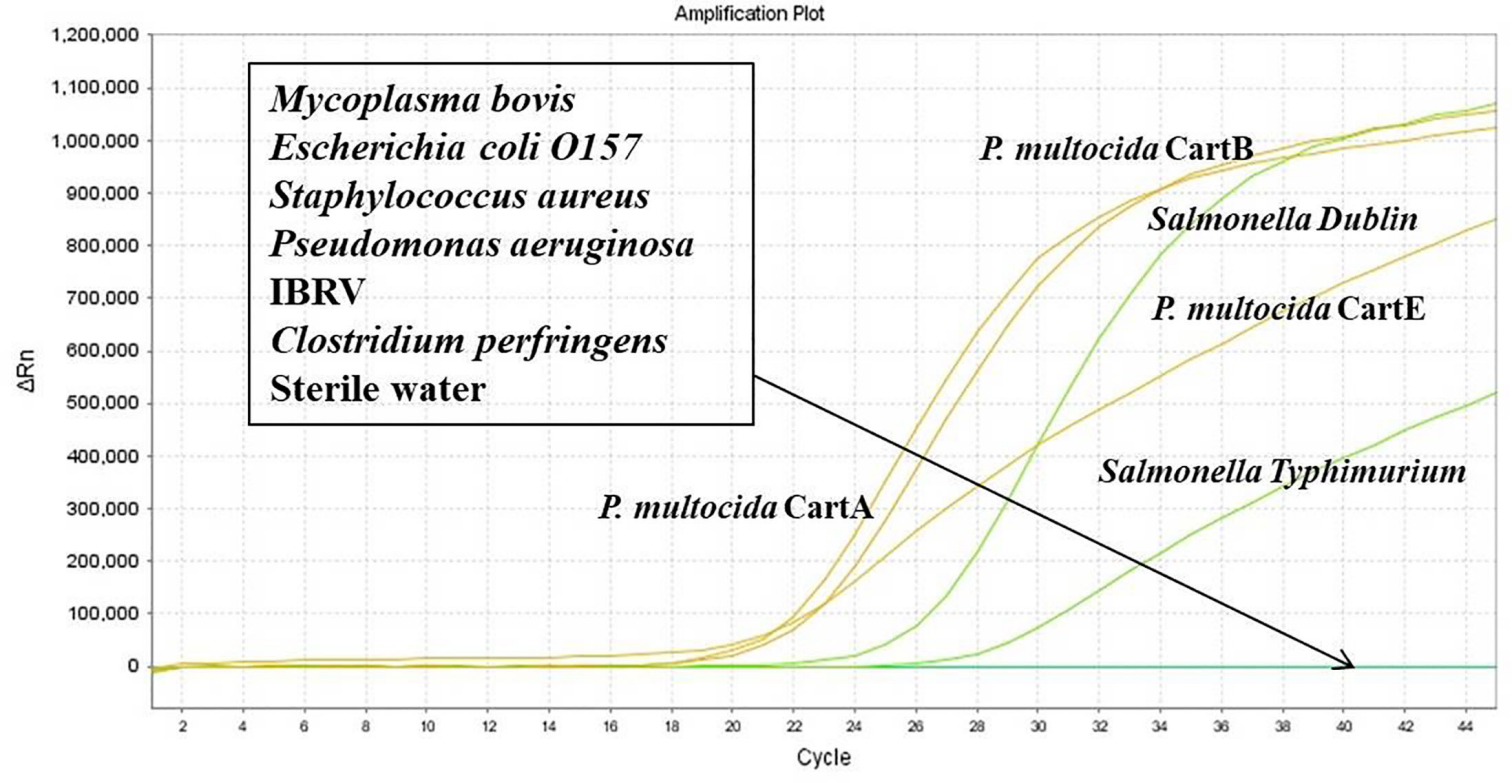
Results of specificity of the dual-fluorescence quantitative PCR method.

As shown in **Figure 5**, using the nucleic acids of *P. multocida* (serotype A, B, and E), *Salmonella Dublin*, and *Salmonella Typhimurium* as templates, fluorescence signals and amplification curves were observed in both the FAM and VIC channels. In contrast, no amplification curves or fluorescence signals were detected for the nucleic acids of non-target pathogens such as *Mycoplasma bovis, Escherichia coli* O157, *Staphylococcus aureus, Pseudomonas aeruginosa*, IBRV, *and Clostridium perfringens*.

### 3.4 Results of the stability and anti-interference tests

As shown in **Table 2**, no interference was detected in the amplification of high-concentration pathogen against the another low-concentration pathogens. After statistical analysis of the Ct values of the dual fluorescence quantitative PCR amplification curves, it was found that the coefficient of variation for Ct values of *P. multocida* and *Salmonella* ranged from 0.23% to 1.51% (**Table 3**).

**Table 2:** Anti interference detection results of dual fluorescence quantitative PCR method.

	*P. multocida*			*Salmonella*		
1	10^8^			10^3^		
Ct value	22.00	22.18	21.89	38.21	38.15	38.09
2	10^3^			10^8^		
Ct value	34.12	34.28	34.08	17.90	17.89	18.01

**Table 3:** Detection results of intra and inter batch of dual fluorescence quantitative PCR method.

Recombinant plasmid	Concentration (copies/μL)	Intra batch testing		Inter batch testing	
		X ± SD	CV (%)	X ± SD	CV (%)
pMD-Pm	10^7^	25.260 ± 0.120	0.48	25.346 ± 0.232	0.91
	10^5^	31.547 ± 0.071	0.23	31.280 ± 0.126	0.40
	10^3^	38.183 ± 0.259	0.68	38.217 ± 0.105	0.27
pMD-SE	10^7^	21.583 ± 0.325	1.51	21.483 ± 0.227	1.06
	10^5^	27.360 ± 0.167	0.61	27.527 ± 0.146	0.53
	10^3^	34.447 ± 0.381	1.11	34.213 ± 0.181	0.52

### 3.5 Determination of cut-off value

**Figure 6:**
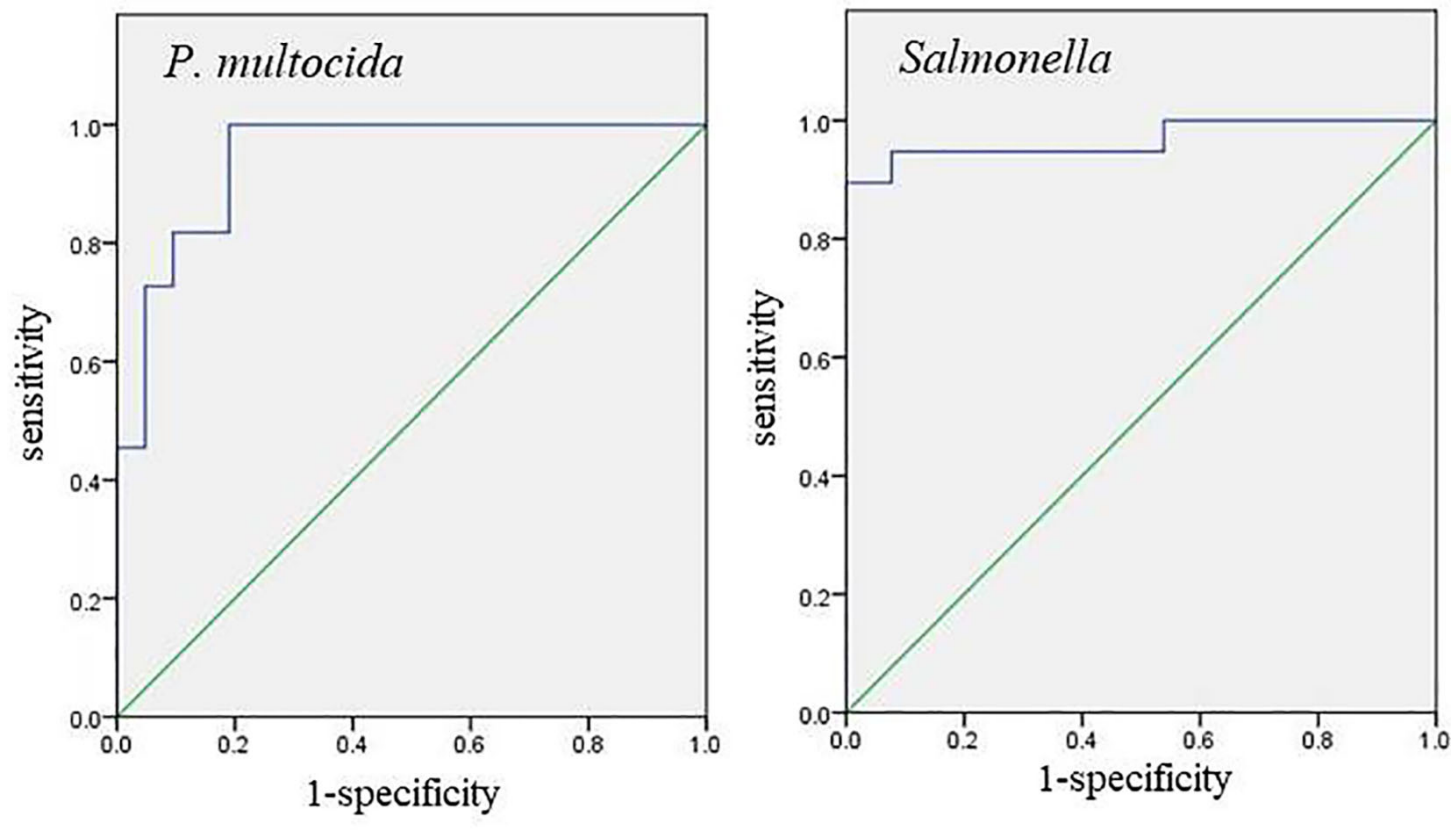
ROC curve analysis of 100 sample test results.

Fifty positive samples and fifty negative samples with known backgrounds were tested. For *P. multocida* and *Salmonella*, the sensitivity values were 1.0 and 0.895, respectively, while the specificity values were 0.81 and 1.0, respectively. The areas under the curve (AUC) were 0.944 and 0.968, respectively (**Figure 6**). The maximum Youden index were 0.81 and 0.895, corresponding to Ct values of 41.0 and 40.5, respectively. The experiment was considered valid when the positive controls for *P. multocida* and *Salmonella* (FAM and VIC) displayed typical S-shaped amplification curves, while the negative controls (FAM and VIC) showed no amplification curves and Ct values ≥45 or no values.

### 3.6 Positive and negative result determination

If a typical S-shaped amplification curve appears in the FAM detection channel and the Ct value is ≤ 41; If the Ct value of the VIC detection channel is ≥ 45 or there is no Ct value, and there is no typical S-shaped amplification curve, it is determined to be *P. multocida*. If a typical S-shaped amplification curve appears in the VIC detection channel and the Ct value is ≤ 40.5; If the Ct value of the FAM detection channel is ≥ 40 or there is no Ct value, and there is no typical S-shaped amplification curve, it is determined to be *Salmonella*. If the critical value of the test is ≤ Ct value < 45, it is considered suspicious and it is recommended to perform a double dose retest. If the retest Ct value is<the critical value of the test, it is considered positive. If the retest Ct value is ≥ the critical value of the test, it is considered negative. If the Ct value is ≥ 45 or there is no Ct value and no typical S-type amplification curve, it is judged as negative for *P. multocida* and *Salmonella*. The results are summarized in **Table 4**.

**Table 4:** Description of judgment results.

FAM	VIC	Judgment
Positive	Negative	Containing *P. multocida* nucleic acid
Negative	Positive	Containing *Salmonella* nucleic acid
Positive	Positive	Containing *P. multocida* and *Salmonella* nucleic acid
Negative	Negative	No nucleic acids of *P. multocida* or *Salmonella*

### 3.7 Detection results of clinical samples

**Figure 7:**
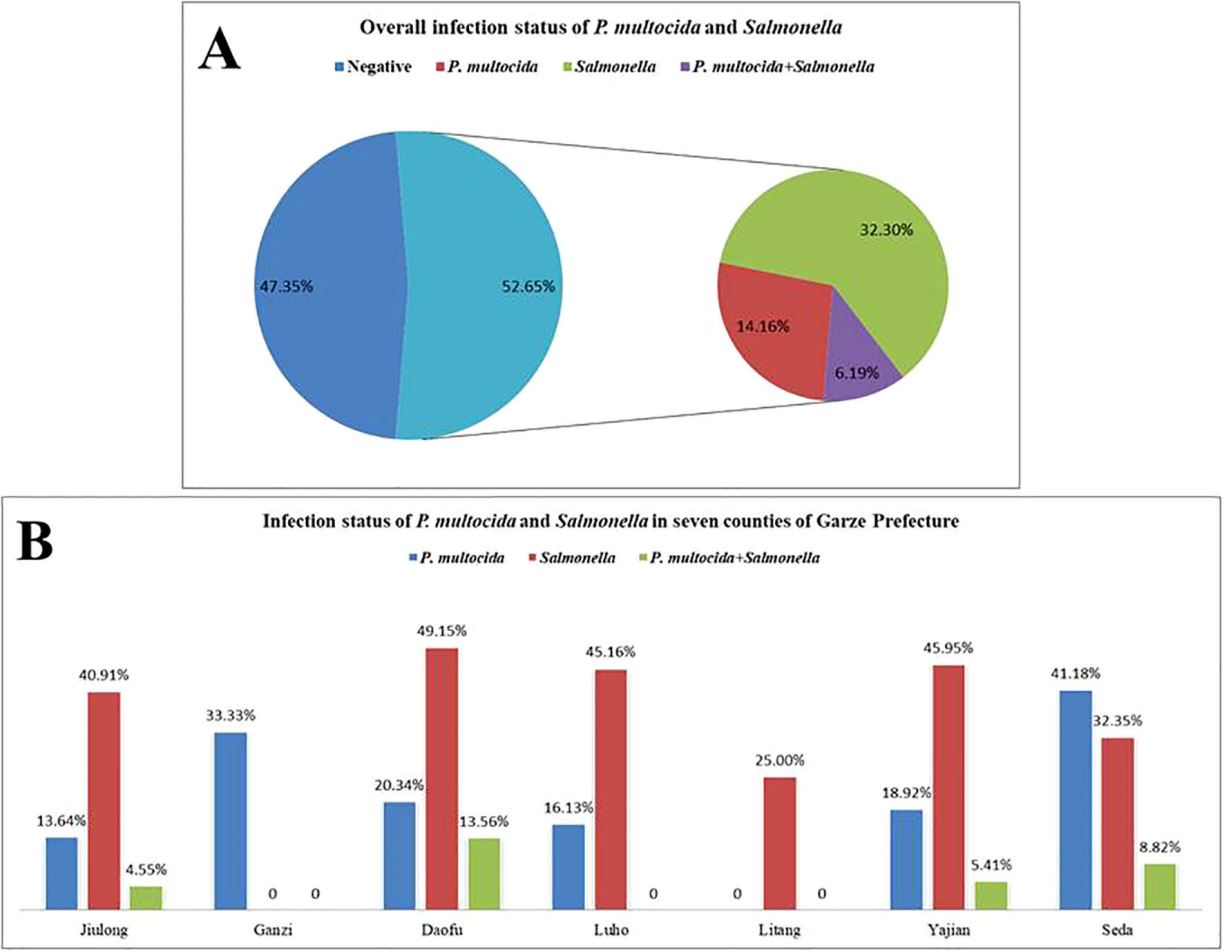
Detection results of clinical samples from seven counties in Ganzi Prefecture. **(A)** Overall infection status of *P.multocida* and *Salmonella*; **(B)** Infection status of *P.multocida* and *Salmonella* in seven counties of Garze Prefecture.

A total of 226 samples were tested using the dual-fluorescence quantitative PCR method established in this study and a previously reported method. The results showed an overall positivity rate of 52.65% (119/226), with a positivity rate of 20.35% (46/226) for *P. multocida*, 38.50% (87/226) for *Salmonella*, and a co-infection rate of 6.19% (14/226). The detailed infection status is presented in **Figures 7A, B**. Additionally, the concordance rate with the previously reported method was 100%.

## 4 Discussion

The yak, a vital economic and ecological resource in high-altitude regions, is essential for local livelihoods and regional sustainability. However, yaks face significant health threats from pathogens like *Pasteurella multocida* and *Salmonella*, which cause respiratory diseases, diarrhea, and hemorrhagic septicemia, resulting in substantial economic losses. Additionally, *Salmonella* poses zoonotic risks, while *P. multocida* may also threaten human health through environmental exposure. Traditional diagnostic methods, such as pathogen isolation and PCR, are limited by low sensitivity, specificity, and throughput. This underscores the need for a sensitive, specific, and high-throughput duplex real-time fluorescence quantitative PCR (qPCR) assay to improve disease diagnosis and control.

In this study, we developed a duplex qPCR assay targeting the species-specific genes *kmt1* of *P. multocida* and *invA* of *Salmonella*. Conserved and specific regions were identified through sequence alignment, and primers and probes were designed accordingly. After optimizing the assay conditions, the assay demonstrated strong specificity, with no cross-reactivity observed with other pathogens, such as *Mycoplasma bovis*, infectious bovine rhinotracheitis virus and others. The assay exhibited high sensitivity, with detection limits of 100 copies for *P. multocida* and 10 copies for *Salmonella*. Compared to the single-plex qPCR assays for *P. multocida* (50 copies) and *Salmonella* (100 copies) reported by Pansri et al (Pansri et al., 2020; Pansri et al., 2022), our method achieved equivalent or superior sensitivity. The assay also displayed excellent repeatability and stability, with intra- and inter-assay variations below 2%. Statistical analysis using SPSS and Receiver Operating Characteristic (ROC) curves determined the critical Ct values for detecting *P. multocida* and *Salmonella* to be 41.0 and 40.5, respectively. Unlike conventional methods, which rely solely on the presence of amplification curves or Ct values to define positive results potentially leading to false positives due to non-specific amplification (Xin et al., 2023; Mengoli et al., 2009; Cruciani et al., 2023). We used statistical methods to ensure greater accuracy in setting positive and negative thresholds.

Epidemiological studies on *P. multocida* and *Salmonella* have been widely reported both domestically and internationally. The prevalence of *P. multocida* in cattle ranges from 8.35% to 33.01% in regions such as Northeast China and Xinjiang (Almoheer et al., 2022; Wang H. et al., 2023; Zhou et al., 2023), with a prevalence of 38.4% reported in Denmark (Goecke et al., 2021). *Salmonella* infection rates in livestock vary from 13% to 33.3%, with *S. Dublin* accounting for 20% of cases (Liu et al., 2022; Wang et al., 2023a; Wang et al., 2023b). In Henan Province, *Salmonella* was recovered from 21.09% (89/422) of raw milk samples (Liu et al., 2022). In California, USA, the infection rate among cattle was reported to be 6.6%, with Salmonella isolates derived from 60% of water buffalo and 64.28% of dairy cattle (Chen et al., 2019; Fatima et al., 2023). In Ganzi Prefecture, Sichuan Province, yak farming is gradually becoming more intensive and large-scale; however, data on the prevalence of *P. multocida* and *Salmonella* in yak populations are limited. In this study, samples were collected from seven counties in Ganzi Prefecture to assess the prevalence of these pathogens. The results showed that the single infection rates for *P. multocida* and *Salmonella* were 20.35% (46/226) and 38.50% (87/226), respectively, with a co-infection rate of 6.19% (14/226). These pathogens were found to be prevalent to varying degrees across all counties. Importantly, the results obtained using the developed duplex qPCR assay were consistent with those from previously reported methods, confirming the reliability and applicability of this assay.

## 5 Conclusion

In this study, we successfully developed a highly sensitive, specific, rapid, duplex real-time fluorescence quantitative PCR assay for the simultaneous detection of *P. multocida* and *Salmonella* in yaks. This method offers robust technical support for the diagnosis and control of *P. multocida*-induced pasteurellosis and Salmonella-associated diseases. Furthermore, the application of this detection method to clinical samples from various counties in Ganzi Prefecture has supplemented the epidemiological data on the prevalence of *Pasteurella multocida* and *Salmonella* in local yak populations. These findings are of significant value for the prevention and control of these diseases, contributing to the sustainable development of yak farming in high-altitude regions.

## Data Availability

The original contributions presented in the study are included in the article/supplementary material. Further inquiries can be directed to the corresponding authors.
